# Motherhood after breast cancer: can we balance fertility preservation and cancer treatment? A narrative review of the literature

**DOI:** 10.5935/1518-0557.20180032

**Published:** 2018

**Authors:** Márcia M Carneiro, Ana M Cota, Maria C Amaral, Moisa L Pedrosa, Bruna O Martins, Marcelo H Furtado, Rivia M Lamaita, Marcia C F Ferreira

**Affiliations:** 1 Centro de Reprodução Humana Hospital MATER DEI, Belo Horizonte-MG; 2 Departamento de Ginecologia e Obstetrícia e Obstetrícia da Faculdade de Medicina da UFMG; 3 Equipe Multidisciplinar de Endometriose Biocor Hospital, Belo Horizonte-MG

**Keywords:** Assisted reproductive technologies, fertility preservation, breast cancer, *in vitro* fertilization, pregnancy, embryo cryopreservation, oocyte cryopreservation

## Abstract

Breast cancer may affect young women who have not yet completed childbearing. Assisted reproductive technology (ART) provides alternatives for fertility preservation such as oocyte, embryo or ovarian tissue cryopreservation. We reviewed the published literature on fertility-preserving management in breast cancer, aiming at finding evidence to answer the following questions: (1) What are the fertility sparing options available?; (2) How do these women respond to IVF? and (3) Can pregnancy influence breast cancer recurrence? There is a paucity of publications describing clinical experience and outcome data which limits accessibility to fertility preservation in this setting. Presently, oocyte or embryo cryopreservation are the main options for fertility preservation. IVF success rates are comparable to the ones of non-oncological populations according to the woman's age but current published studies lack data on definitive success rates following embryo banking for cancer patients. The perception that IVF and pregnancy may worsen cancer prognosis remains, despite the lack of scientific evidence to support this notion. Published studies show reassuring results for pregnancies occurring >2 years after breast cancer diagnosis. The best published evidence suggests pregnancy after breast cancer does not increase the risk of disease recurrence, thus pregnancy should not be forbidden once treatment is completed. Decision making for women diagnosed with cancer requires up-to-date knowledge of the efficacy and safety of available options. Providing consultation with a reproductive specialist and appropriate information on fertility preservation for these women should be an essential aspect of their supportive care.

## INTRODUCTION

Cancer still represents an enormous global health burden, and published data revealed about 14.1 million new cases and 8.2 million deaths in 2012 worldwide (Torre *et al*., 2016). Cure remains the most important therapeutic target, and current available therapies are based on surgery, cytotoxic medications and/or radiation, which in turn could unfortunately result in partial or total loss of fertility.

The availability of new treatment modalities has improved cancer survival rates over the last two decades, putting quality-of-life issues in the spotlight for women who survive the disease. Fertility care is a growing issue in this setting (Jeruss & Woodruff, 2009; Rowan, 2010). The development of assisted reproductive technology (ART) and cryopreservation techniques, provided alternatives for female fertility preservation such as oocyte, embryo or ovarian tissue freezing. Temporary ovarian suppression with GnRH analogues during chemotherapy is also an option in this setting (Rowan, 2010; von Wolff *et al*., 2015; Lambertini *et al*., 2015; Lambertini *et al.,* 2016).

Breast cancer is the most common cancer in women, and in 2017 the American Cancer Society (ACS) estimates that there will be 252,710 cases of invasive breast cancer diagnosed in US women and 40,610 deaths. Data also shows that breast cancer is responsible for 30% of new cancer cases, and 1 in 8 women will develop breast cancer during their lifetime (Smith *et al*., 2017). Between 2014 and 2015 the National Cancer Institute (INCA) in Brazil expected that 57120 new cases of breast cancer would be diagnosed with an estimated risk of 56.09 cases in every 100,000 women.

The risk of developing breast cancer increases with age and 6 to 10% of the cases occur in women under 40 years of age. Approximately, 215.8 per 100,000 women will be diagnosed with breast cancer at age ≤44 years (Banz-Jansen *et al*., 2013). These young women deserve proper evaluation and counseling in order to adequately evaluate risks and benefits of treatments. As breast cancer may affect young women who are still in their reproductive years and many are postponing childbearing, the incidence of cancer in those who still want to get pregnant has somewhat increased. Many factors may affect rates of permanent infertility and compromised fertility after cancer treatment (Banz-Jansen *et al*., 2013; Lambertini *et al*., 2016). The effects of chemotherapy and radiation therapy on fertility depend on a number of factors: the drug or size/location of the radiation field, dose, dose-intensity, method of administration, disease, age, sex, and pretreatment ovarian reserve and parity (Salama *et al*., 2013; Lawrenz *et al*., 2016).

Recent improvements in the prognosis of cancer patients has drawn the attention to fertility issues. Safe conservative options that preserve fertility are available and may be adopted for those who have not completed their childbearing potential (Rowan, 2010; Levine *et al*., 2015; Druckenmiller *et al*., 2016; Fournier, 2016). Research on new methods such as *in vitro* follicle maturation and techniques for tissue transplantation is ongoing (Loren *et al.*, 2013).

The FIGO Committee for the Ethical Aspects of Human Reproduction and Women's Health advises that cancer treatment is the primary goal. The risks of delaying treatment in order to induce ovarian stimulation and retrieval, ovarian removal or transplant must be carefully considered and should not have a significant impact on treatment (FIGO, 2012). Information on fertility preservation options is mandatory in such a context (Loren *et al*., 2013; Tomasi-Cont *et al*., 2014; Fournier, 2016).

## METHODS

We set out to perform a literature narrative review on breast cancer and fertility preservation. We searched in PubMed up to July 2017 for relevant papers without language restriction. The following keywords were used: "fertility preservation", "breast cancer", "*in vitro* fertilization", "pregnancy", "embryo cryopreservation", "oocyte cryopreservation", "ovarian tissue cryopreservation", "gonadotropin hormone-releasing hormone analogs".

Initially, the PubMed search revealed 665 papers, out of which 417 abstracts were selected. After reading the abstracts, 86 full-text articles were obtained and finally 61 papers were included in the review. The search proved to be difficult, since much of the data on breast cancer was mixed with other types of female cancer, as well breast cancer diagnosed during pregnancy or spontaneous pregnancy after breast cancer. Articles on the risk of breast cancer after controlled ovarian stimulation and assisted reproduction, and papers related to gynecological malignancies in general and review articles were excluded. Whenever possible, we collected data on breast cancer only. We included only articles reporting data on breast cancer and fertility preservation strategies: medical treatment and assisted reproductive technologies, including IVF and controlled ovarian stimjulation. We also included guidelines pertaining to the management of breast cancer and fertility preservation in gynecological cancers.

**Figure 1:**
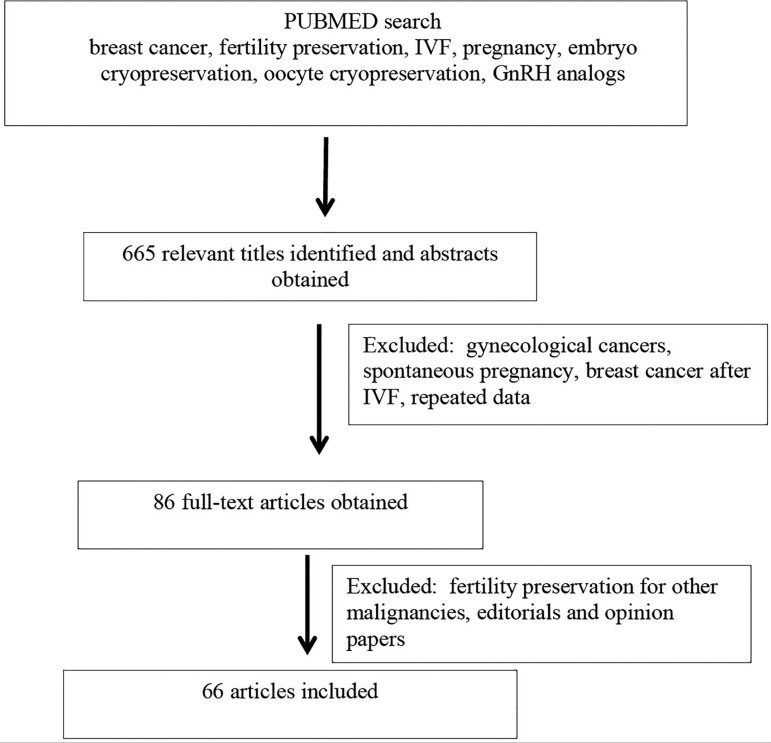
Flow chart showing revision process

After reading the full texts, 66 papers were selected (Figure 1). No randomized clinical trials were available on the use of fertility-sparing treatment in breast cancer, and the majority of the publications were case series reports. Guidelines of the American Society for Reproductive Medicine(ASRM), European Society for Medical Oncology (ESMO); National Comprehensive Cancer Network (NCCN), The Society of Obstetricians and Gynaecologists of Canada(SOCG) and the International Federation of Gynecology and Obstetrics (FIGO) were also taken into account.

We reviewed the published literature about safe fertility-preserving management in breast cancer, focusing on the selection criteria of the patients, available treatment options and follow-up. We focused on finding evidence to answer the following relevant clinical questions:

1. What are the fertility sparing options available?
2. How do these women respond to IVF?
3. Can pregnancy influence breast cancer recurrence?

## RESULTS

### 1. What are the fertility sparing options available?

Studies have shown that, despite being a major concern of most young patients diagnosed with breast cancer, fertility risks and information about fertility preservation techniques have only been disclosed to the majority of them (68%), but only a small part of the patients (10%) used fertility preservation (Bastings *et al*., 2014; Ruddy *et al*., 2014).

To respond to these patients' expectations, the assessment of ovarian reserve should guide the physician in counseling cancer patients about expected success with fertility preservation techniques. Presently, the woman's age is the single most important predictor for success with artificial reproductive techniques, with pregnancy rates declining with advancing age (Cil *et al.,* 2013). Other forms of evaluating ovarian reserve, such as early follicular phase follicle-stimulating hormone, anti-Mullerian hormone, and antral follicle count, are predictive of the number of oocytes retrieved with ovarian stimulation, and are associated with pregnancy rates (ASRM, 2012).

Available techniques for fertility preservation include ovarian suppression, oocyte and embryo cryopreservation, immature oocyte retrieval, *in vitro* maturation and ovarian tissue cryopreservation. At the present moment, the main option for fertility preservation is oocyte or embryo cryopreservation. It was considered standard by the American Society of Reproductive Medicine in 2013. As the technique requires ovarian hyperstimulation, it should be considered at diagnosis, before initiating systemic treatment. In fact, counselling with a fertility specialist can optimize the implementation of a fertility-sparing strategy without delaying the cancer treatment.

#### Gonadotropin-Releasing Hormone Analogues

Chemotherapy seems to be most harmful to the ovarian reserve in older women, as they are more prone to have amenorrhoea afterwards. Prepuberal girls appear to have less compromised ovarian function after chemotherapy, indicating that ovarian suppression may confer some degree of protection (Ben-Aharon *et al*., 2010; Levine *et al*., 2015).

Experimental studies in animals have demonstrated a protective effect; however, studies with women have shown conflicting results (Ben-Aharon *et al*., 2010; Gerber *et al*., 2011; Bedaiwy *et al*., 2011). The most widely studied suppression method uses gonadotropin releasing hormone agonists (GnRHa). A metanalysis showed that cotreatment with GnRH agonists can protect patients from post-chemotherapy ovarian failure, but does not have an effect on preserving fertility (Sun *et al*., 2014).

Medical societies' guidelines have stated that there is not conclusive evidence that GnRH analogues are really effective in proctecting ovarian function from chemotherapeutic agents (ASRM, 2013; Loren *et al*., 2013). Recently, however, a meta-analysis including 12 randomized controlled trials with 1231 breast cancer patients revealed that ovarian suppression with GnRHa in young women with breast cancer reduced the risk of ovarian failure after chemotherapy. Pregnancy rates were increased without adverse effects on cancer prognosis (Lambertini *et al*., 2015). It is important to point out that the possible effect of GnRH in protecting ovarian reserve can only be assessed after ending chemotherapy.

#### Immature Oocyte Cryopreservation

Another method of fertility preservation is to retrieve immature oocytes transvaginally, without hormonal stimulation. This procedure is followed by *in vitro* maturation and cryopreservation of mature oocytes or even embryos. This technique presents advantages such as the possibility of obtaining oocytes with no hormonal hyperstimulation, and no delay to start oncological therapy. It also ensures that estrogen levels are kept in the physiological range and has limited costs compared to cryopreservation of mature oocytes following ovarian stimulation (Levine *et al*., 2015; de Pedro *et al.,* 2015).

The main drawback, however, is that success rates are lower than the ones obtained with cryopreservation of oocytes or embryos that have matured *in vivo*, and it is still considered experimental (Levine *et al*., 2015; de Pedro *et al*., 2015).

#### Ovarian Tissue Cryopreservation

This experimental procedure, that has received considerable attention, consists of laparoscopic oophorectomy or ovarian tissue biopsy, followed by ovarian cortical tissue dissection into small fragments and cryopreservation. Similarly to the retrieval of immature oocytes, no ovarian stimulation is required, and there is minimal delay in treatment. Also, no partner is needed. This is the only available option for prepubertal children (Donnez *et al*., 2004; de Pedro *et al*., 2015).

After oncological treatment, tissue can be transplanted or follicles can be aspirated, and the oocytes are matured *in-vitro*. There are several reported live births using this techinque and orthotopic autologous transplantation (Donnez *et al*., 2013).

Although there have been no reported cases of recurrent cancer after transplantation in humans, there is concern that transplanted ovarian tissue could be contaminated with cancer cells. This is mainly a concern for BRCA mutation carriers, leukemias and tumors that involve the ovaries (Bastings *et al.,* 2013).

#### Ovarian stimulation and cryopreservation of mature oocytes or embryos

It is considered the safest option for preserving fertility in patients with cancer. Controlled ovarian stimulation (COS) is achieved by subcutaneous injection of gonadotropins for 8 to 14 days, along with pituitary blockage with GnRH analogues. Follicular growth is monitored by transvaginal ultrasound and final oocyte maturation can be triggered by hCG or GnRH-a. However, due to the concerns pertaining to the effects of supraphysiological hormonal concentrations, several different protocols have been studied in an attempt to minimize possible worsening in oncological prognosis (Azim *et al*., 2008; de Pedro *et al*., 2015).

Tamoxifen, a selective estrogen receptor modulator, with proven effects in reducing mortality and relapse rates in patients with breast cancer, did not interfere with the number of oocytes retrieved during controlled ovarian stimulation (Meirow *et al*., 2014).

Aromatase inhibitors have also shown to decrease estrogen serum levels in postmenopausal women with breast cancer, and they are also effective in reducing mortality and relapses in breast cancer. The use of letrozole was proven safe in patients undergoing COS (de Pedro *et al.,* 2015), and it is recommended to reduce estrogen concentration without a decline in oocyte yield (Muñoz*et al*., 2015; Rodgers *et al*., 2017).

The use of GnRHa as an alternative to hCG in antagonist protocol cycles is now established as an alternative to reduce the likelihood of the patient developing ovarian hyperstimulation syndrome, without negative effects on the number of oocytes collected and their maturation status. It is thought to be beneficial in patients with breast cancer, by enabling rapid reduction of estradiol levels after oocyte retrieval (Rodgers *et al*., 2017).

Ocyte retrieval is performed under sedation and the mature oocytes collected are cryopreserved. If the patient wishes to do so, the oocytes can be fertilized with the partner or donor sperm, and the resulting embryos can be cryopreserved. After the cancer treatment is finished and the patient is cleared by the oncologists to get pregnant, thawed oocytes are fertilized and the embryos are then transferred to the uterus. Those who carry genetic mutations for familial cancers may also be candidates for pre-implantation genetic diagnosis to select unaffected embryos, and thus avoid passing the mutation to offspring (Oktay *et al*., 2015; Shapira *et al*., 2015).

Success rates are comparable to those of non-oncological populations for IVF, and may vary according to the woman's age. Published data reveals a 42% live birth rate per thawed embryo transfer in women <35 years of age, 40% in women who were 35 to 37 years old, and 34% in women who were 38 to 39 years old (Muñoz *et al*., 2015; CDC, 2014; Oktay *et al*., 2015). Table 1 summarizes the advantages and disadvantages of the available fertility preservation techniques.

**Table 1:** Fertility preservation techniques.

Technique	Advantage	Disadvantage	Practice
IVF and embryo cryopreservation	Most effective	COS Requires partner or donor Requires time for stimulation	Standard
Mature oocyte cryopreservation	Effective Does not require partner or donor	COS Requires time for stimulation Few pregnancies reported	Standard
Immature oocyte cryopreservation and *in vitro* maturation	No delay in treatment No COS Does not require partner or donor	Few pregnancies reported	Experimental
Ovarian cortex cryopreservation	No COS No delay in treatment Suitable for prepubertal girls	Requires surgery Few pregnancies reported Potential risk of cancer grafts	Experimental
Ovarian supression with GnRH	No COS No delay in treatment Non invasive	Effectiveness not proven Climateric symptoms	Not proven

COS:Controlled ovarian stimulation

### 2.How do these women respond to IVF?

As previously discussed, IVF with controlled ovarian stimulation (COS) and embryo or oocyte cryopreservation is the most effective option for fertility preservation in breast cancer. Ovarian stimulation significantly increases estradiol levels, which raises concerns regarding safety of such a procedure as well as the possible role of malignancy and BRCA mutation in reducing ovarian response to stimulation (Shapira *et al*., 2015).

The addition of aromatase inhibitors to ovarian stimulation is a strategy which has been successfully used in breast cancer patients to reduce estradiol levels during stimulation (Oktay *et al*., 2005; Azim et al., 2008; Oktay *et al*., 2015). The safety of performing COS using Letrozole in young women with breast cancer before chemotherapy has been evaluated. After a 5-year follow up, 120 young breast cancer patients who underwent COS had comparable survival and recurrence rates to the 217 who did not undergo COS (Kim *et al*., 2016).

Letrozole has been used in COS to suppress estradiol levels without significantly impacting oocyte yield or reducing disease-free survival rates. They caution that the safety of COS in women with breast cancer derives from a small number of observational studies. Unfortunately high quality evidence is difficult to come by due to ethical and practical reasons (Rodgers *et al*., 2017).

Protocols with different timing to begin COS have been developed in order to expedite treatment. It may be possible to perform two consecutive ovarian stimulation cycles with the use of letrozole-gonadotropin protocol for these women without further delaying initiation of cancer therapy (Turan *et al*., 2013). In an attempt to maximize the number of retrieved oocytes without delaying oncologic treatment, a new ovarian stimulation protocol (DuoStim) has been developed. This entails two successive ovarian stimulation cycles and two oocyte retrievals, but it has been used in only 10 patients so far (Tsampras *et al*., 2017).

Many studies that have been performed to evaluate ovarian performance in women with cancer present controversial results. These publications involve women with diferent types of cancers undergoing IVF, the majority of them suffering from breast cancer (Shapira *et al*., 2015). Only two studies reported worse oocyte yield in comparison to women without cancer undergoing IVF (Klock *et al*., 2010; Domingo *et al*., 2012). Quinn *et al.* (2017) published a retrospective cohort analysis with 589 women (191 with breast cancer) who underwent COS. The group with breast cancer responded as well as the ones without cancer in terms of number of mature oocytes obtained.

Another study evaluated the use of letrozole and gonadotropins in women with breast cancer undergoing COS for elective cryopreservation of oocytes. These women obtained more oocytes (12.3±3.99) in comparison to the elective cryopreservation group (10.9±3.86; *p*<0.01), as well as comparable live-birth-rates (32% x 39.7%, respectively) (Pereira *et al*., 2016).

Although success rates are comparable to the ones of non-oncological populations for IVF and may vary according to the woman's age, current published studies lack data on definitive success rates following embryo banking for cancer patients. There are only a handful of reports based on small series which present reassuring live born rates in cancer patients who have undergone thawed embryo transfer (Ben-Haroush *et al*., 2011; Goldrat *et al*., 2015; Luke *et al*., 2016). No data regarding embryo quality has been published so far.

Large studies including women seeking fertility preservation before undergoing breast cancer treatment are not available. Results of a large population-based study involving more than 53,000 women treated with ART within 5 years after cancer diagnosis, revealed that women with cancer pursue such treatments at a younger age than those without cancer. Apparently, breast cancer, cervical cancer and all female genital cancers were associated with reduced pregnancy and live birth rates after ART. Prior cancer diagnosis did not influence live birth rates per donor oocyte, but a reduction was found in those using autologous oocytes. This may be explained by pre or periconceptional events which could adversely affect pregnancy rates (Luke *et al*., 2016).

### 3. Can pregnancy influence breast cancer recurrence?

Approximately 50% of premenopausal women with a history of breast cancer will desire a future pregnancy. Unfortunately only 4 to 7% will get pregnant. One explanation is the impact of breast cancer and its treatment on female fertility. Another reason is that both patient and physician dread a negative impact of pregnancy on the control and prognosis of breast cancer (Raphael *et al*., 2015; Litton, 2012).

The safety of pregnancy after breast cancer is uncertain. Available published studies on the impact of pregnancy on the prognosis of breast cancer suggest that women who become pregnant after having breast cancer have a better overall survival when compared with women who did not. Mueller *et al.* (2003) and Velentgas *et al*. (1999) found that women who become pregnant after breast cancer treatment have a lower risk of death when compared to women who did not (RR 0.54 and 0.8, respectively), and this is significantly lower in women younger than 35 years of age (Mueller *et al.,* 2003; Velentgas *et al*., 1999). Similar results were found by Blakely *et al.* (2004), who reported that pregnancy after breast cancer treatment did not increase the risk of recurrence or death. A large meta-analysis of 14 studies showed that pregnancy after a breast cancer lowered the risk of death by 41% (Azim *et al*., 2011). However, the reduced risk of death could be attributed by a selection bias known as "healthy mother effect". Apparently, women who became pregnant after breast cancer treatment felt healthier and thus had better prognosis than the ones who did not become pregnant. Another large meta-analysis addressed the same subject and tried to overcome the bias of the healthy mother effect. After considering the potential for such a bias in the matched controls, ten studies were eligible, and nine contained data appropriate for analysis. Overall survival was statistically higher among patients who became pregnant than among those who did not, showing that pregnancy occurring at least 10 months after a breast cancer diagnosis does not jeopardize prognosis and might even confer a significant survival benefit (Valachis *et al*., 2010). The same results were also recently reported in a third meta-analysis studying the safety of pregnancy after surgical treatment for breast cancer. No increase in breast cancer recurrence rate was observed, and a possible improvement in outcome (overall survival) was also reported (Luo *et al.*, 2014).

The impact of pregnancy on breast cancer prognosis according to hormone receptor status is another source of debate. Azim *et al*. (2013) analysed the impact of pregnancy on disease-free survival in women with a history of breast cancer according to estrogen receptor status. Apparently, pregnancy after estrogen receptor positive tumors did not appear to reduce the risk of recurrence.

Physicians still debate how long women should wait to get pregnant after a breast cancer diagnosis and treatment. Some cohort studies suggest that the survival rates would be better if women delayed pregnancy for 2 years or more after breast cancer treatment (Ives *et al*., 2007). Nye *et al.* (2017) on the other hand did not find reduced disease-free survival for premenopausal women with estrogen receptor-positive breast cancer who became pregnant within 5 years of the diagnosis. Published studies show reassuring results for pregnancies occuring >2 years after breast cancer diagnosis, as well as for the possible adverse effects of pregnancy and high incidence of tumour recurrence during the first 2 years. Therefore, a minimum period of 2 years following diagnosis is advisable before attempting to get pregnant (Azim *et al*., 2011; Peccatori *et al*., 2013).

Goldrat *et al*. (2015) were the first to study the effect of using ART on recurrence and death rates in 198 women who were previously treated for breast cancer and became subsequently pregnant. They attempted to assess the association between ART use and clinico-pathological characteristics, pregnancy outcome and long-term breast cancer outcome. More than 50% of the cases had an endocrine sensitive disease. Full term pregnancies were obtained in 77% and 76% of the spontaneous and ART groups, respectively. After more than 50 months of follow up they found no difference in breast cancer outcome between the two groups.

The European Society for Medical Oncology (ESMO) considers that evidence on any difference in prognosis between pregnant and nonpregnant women with breast cancer is lacking, and it does not recommend pregnancy termination regardless of tumor status (Peccatori *et al*., 2013). The Society of Obstetricians and Gynecologists of Canada (SOGC), with a low level of evidence, recommends that women wait at least 3 years before attempting pregnancy and 5 years if there is nodal involvement (Helewa *et al.,* 2002). Those timeframes are quite difficult in terms of fertility maintenance after breast cancer treatment. Current available guidelines are summarized in Table 2.

**Table 2:** International guidelines pertaining to breast cancer and pregnancy.

Guideline	Pregnancy-associated breast cancer	Pregnancy after breast cancer
ESMO 2013	No recommendation for abortion (lack of evidence)	No recommendation against pregnancy (a)
NCCN 2014	No recommendation for medical abortion (discussion in a multidisciplinary setting, discussion with patient)	No recommendation against pregnancy
SOCG 2002	No recommendation for abortion (b)	No recommendation against pregnancy no detrimental effect) (c)

a "Do not discourage pregnancy following breast cancer diagnosis irrespective of the [estrogen receptor] status."

b "In early pregnancy, the patient should be counseled regarding the effects of the proposed therapy on the fetus and on overall maternal prognosis. Termination of pregnancy should be discussed, but the patient should be counseled that prognosis is not altered by pregnancy termination."

c "Woman treated for [breast cancer], who wish to become pregnant should be counseled that pregnancy is possible and does not seem to be associated with a worse prognosis. However, they should be made aware that the evidence to support such advice is relatively poor." ESMO = European Society for Medical Oncology; NCCN = National Comprehensive Cancer Network; SOCG = The Society of Obstetricians and Gynaecologists of Canada.

Overall, the literature is reassuring and does not show a worse outcome for women with previously diagnosed and treated breast cancer who seek to become pregnant afterwards. Some data even suggest a better survival outcome. Those findings should bring comfort to physicians and to women with a previous breast cancer diagnosis.

In summary, the best available published evidence so far suggests that pregnancy after breast cancer does not increase a woman's risk of disease recurrence. Pregnancy should not be forbidden after breast cancer treatment solely because of concerns on cancer recurrence and death, since current available data is rather reassuring. If pregnancy is an option, these women must receive carefully coordinated multidisciplinary approach. More large randomized prospective trials are nedded to develop appropriate protocols in this setting.

## CONCLUSION

Hundreds of thousands of women in their reproductive years are diagnosed with cancer each year. Advances in breast cancer treatment result in increased numbers of female patients who survive cancer raising the demand for effective and individualized fertility preservation options. Unfortunately fertility counseling remains a secondary issue for many breast cancer specialists. The perception that IVF and pregnancy may worsen cancer prognosis remains, despite the lack of scientific evidence to support this notion. Currently there are limited clinical options for fertility preservation, and the paucity of publications describing clinical experience and outcome data has limited accessibility to these options. Decision making for patients diagnosed with cancer requires up-to-date knowledge of the efficacy and safety of available techniques. Providing consultation with a reproductive specialist and appropriate information on fertility preservation for women with breast cancer should be an essential aspect of their supportive care.
